# An Interesting Case of Mixed Dust Pneumoconiosis With Progressive Massive Fibrosis and Cor Pulmonale in a South American Farmer

**DOI:** 10.7759/cureus.28436

**Published:** 2022-08-26

**Authors:** Dina Alnabwani, Ankita Prasad, Nagapratap Ganta, Andrea C Marin, Sharon Hechter, Sandeep Pavuluri, Kajal Ghodasara, Varun Vankeshwaram, Ghadier Alsaoudi, Chirag Patel, Gustavo E Delaluz, Pramil Cheriyath

**Affiliations:** 1 Internal Medicine, Hackensack Meridian Ocean Medical Center, Brick, USA; 2 Medicine, Zaporozhye State Medical University, Zaporozhye, UKR; 3 Internal Medicine, Jersey Shore University Medical Center, Neptune, USA; 4 Allergy and Immunology, Hackensack Meridian Ocean Medical Center, Brick, USA; 5 Pulmonary and Critical Care Medicine, Hackensack Meridian Ocean Medical Center, Brick, USA

**Keywords:** farm dust, mixed dust fibrosis, lung opacity, macules, nodules, dust exposure, cor pulmonale, agriculture, progressive massive fibrosis, mixed dust pneumoconiosis

## Abstract

Pneumoconiosis is an occupational disease found in workers with environmental exposure to organic and inorganic dust, as in mining, sandblasting, pottery, stone masonry, and farming. The inflammatory response of the lung to respirable dust causes the formation of macules, nodules, and fibrosis, and higher silica content in inhaled dust is associated with increased fibrosis. Mixed dust pneumoconiosis (MDP) is characterized by exposure to dust containing 10-20% silica, and its lung imaging show irregular opacities. Histopathology plays a vital role in the diagnosis of MDP. Though it has a favorable outcome, it evolves slowly over many years of constant exposure and is characterized by worsening dyspnea and cough gradually progressing to cor pulmonale. The only effective treatment is removing exposure, which makes it essential to recognize the disease early for a favorable outcome. We present a case of mixed dust pneumoconiosis in a farmer from South America who had asthma. He presented with worsening dyspnea and multiple nodules in both lungs on imaging and cor pulmonale. An extensive workup was done, and it ruled out any malignancy and tuberculosis. Analysis of video-assisted thoracoscopic surgery (VATS) biopsy samples confirmed the diagnosis of mixed dust pneumoconiosis. He had a confluence of irregular nodes in the upper lobes of the lungs, and the largest was 2.1 cm. This fits the International Labour Organization (ILO) definition of progressive massive fibrosis. This, along with cor pulmonale present in him, gives it a poor prognosis even after he is removed from dust exposure. He received steroids, which led to symptomatic improvement, and he was discharged to follow up with the pulmonologist.

## Introduction

Pneumoconiosis means "dust in the lung," referring to interstitial lung diseases related to the inhalation of dust [1]. It typically evolves over many years of exposure. The effect of dust particles depends on the type of dust and the host response. Diffuse interstitial fibrosis, macules, nodules, and massive fibrotic lesions, also known as progressive massive fibrosis (PMF) or complicated pneumoconiosis, are associated with mineral dust exposure. Nodules are the most common lesions following exposure to silicate minerals and dust mixtures containing less than 20% free silica. These lesions vary from a few millimeters to a centimeter or more and have a propensity for the upper lung zones. Soil is representative of minerals found in the earth's crust depending on the local geology. In addition to naturally occurring minerals, soil contains mineral dust and organic materials derived from farming activities. Studies have documented that respirable dust concentrations up to 5 mg/m^3^ are commonly observed around agricultural equipment and during harvesting and soil preparation [2]. Studies also show agriculture dust's cytotoxicity to be more potent than crystalline silica in activating transcription factors (AP-1 and NF-kB), reactive oxygen species generation, such as H_2_O_2_, and hydroxyl radicals, indicative of their ability to induce oxidative stress [2]. In addition, the silica contained in agricultural dust is often 10 to 20% and is fibrogenic [2]. Pneumoconiosis in farm workers is associated with many other lung diseases, like chronic obstructive pulmonary disease, asthma, hypersensitivity pneumonia, and cancer [3].

## Case presentation

**Figure 1:**
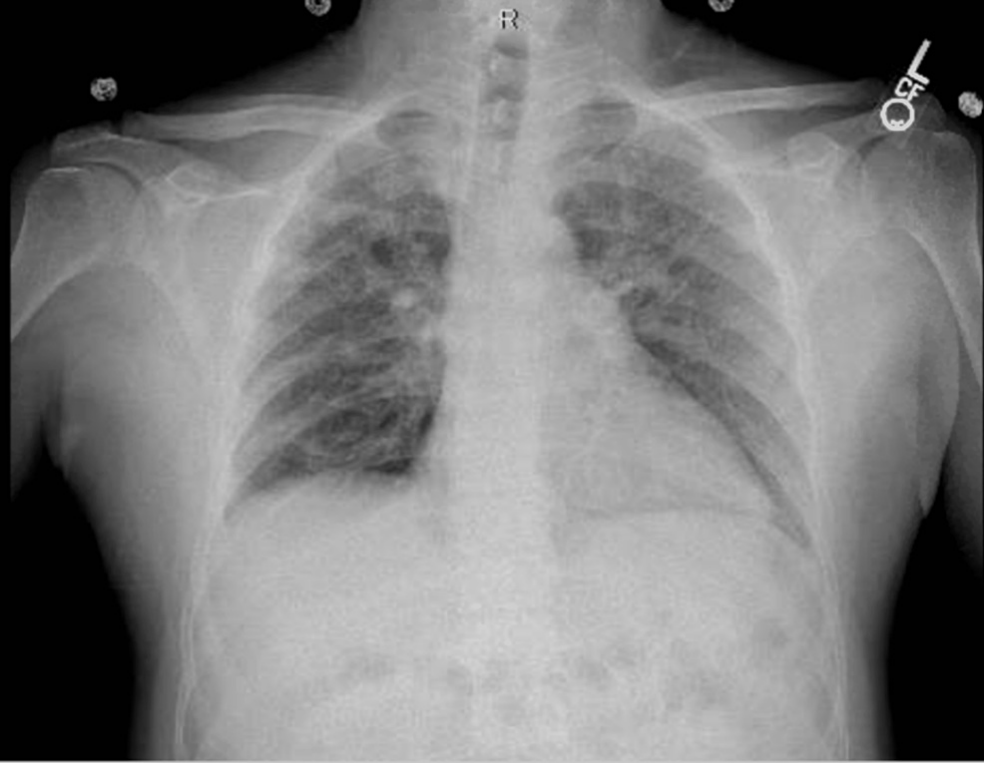
Chest x-ray posteroanterior (PA) view showing fibrosis and nodularity

A 47-year-old male with a history of asthma presented to the emergency room with complaints of progressively increasing fatigue and shortness of breath for two months, but in the last two weeks, the shortness of breath has increased significantly, and it was difficult for him to go up even four to five steps without getting severely dyspneic. He cannot breathe when lying flat and must get up to breathe. His food intake decreased because he felt as if food got stuck in his throat. He had no chest pain, cough, or fever. He is a nonsmoker and nonalcoholic. He reportedly went to a clinic and was told that his electrocardiogram (ECG) showed that he had a heart attack and was told to go to the emergency room. The patient recently migrated from South America to the United States. He was a full-time farmer and had significant exposure to pesticides and chemicals. He had a history of hematemesis eight years ago. At that time, he had an endoscopy, but not much was visualized on endoscopy because of blood. He was asked to follow-up for a repeat endoscopy but could not because of civil unrest in his country. He did not have any further episodes. At the presentation, he was dyspneic, his heart rate was 88/min, his blood pressure was 109/76 mm Hg, SpO_2_ at room air was 97%, and respiration rate was 18/min. He had no pallor, cyanosis, or palpable lymph nodes, but pitting edema was present on his legs. Chest and cardiac examination were normal except for increased work of breathing. ECG showed nonsignificant ST changes. His troponin levels were normal, complete blood count, liver function test, and renal function tests were normal. His echocardiogram shows increased right ventricle (RV) size with pulmonary hypertension (HTN), marked RV pressure overload with normal ejection fraction, and no significant valvular heart disease. A cardiology evaluation and computed tomography (CT) angiography showed no evidence of pulmonary embolism. Right heart catheterization indicated elevated pulmonary capillary wedge pressure (PCWP) and moderate pulmonary HTN with no reversibility with vasodilators. On chest x-ray (CXR), he had multiple pulmonary nodules throughout both lung fields, suspicious of neoplastic and metastatic disease or sarcoidosis (Figure 1).

**Figure 2:**
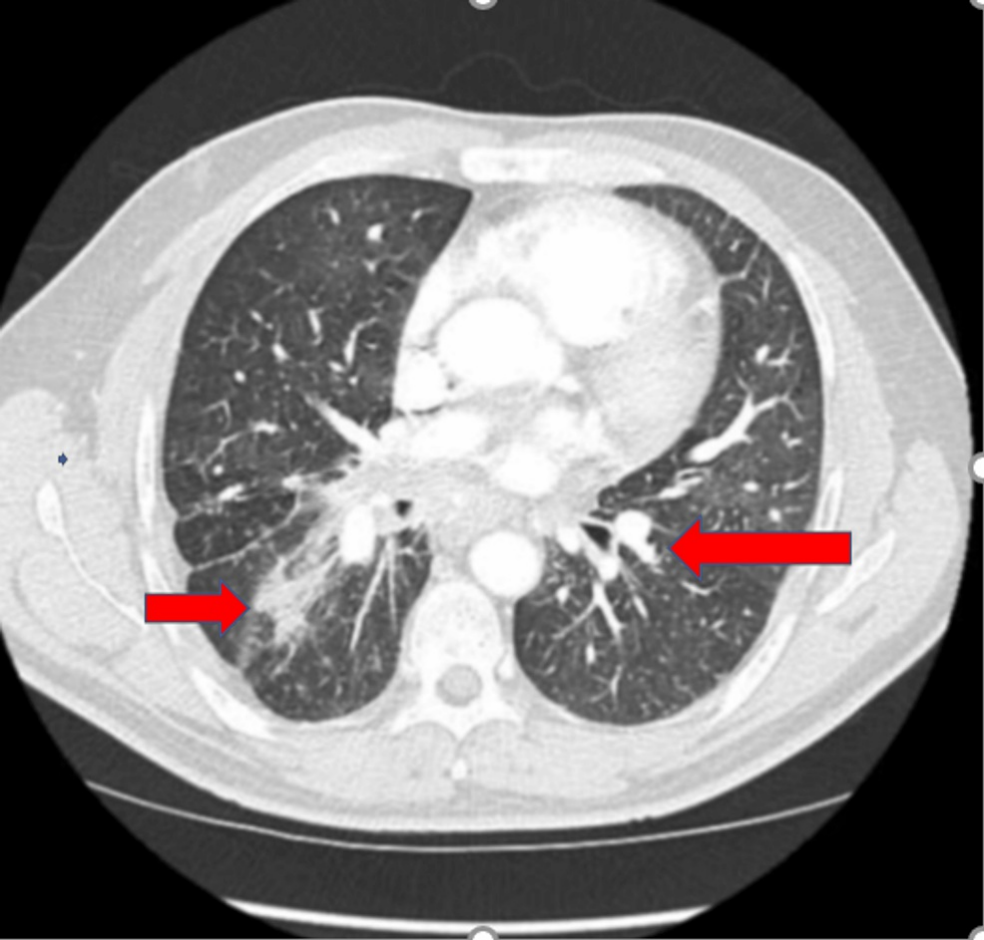
CECT chest transverse view (red arrows) shows nodules with the extensive fibrosis CECT: contrast-enhanced computerized tomography

A rheumatological evaluation ruled out any autoimmune diseases. CT of the chest and abdomen showed multiple bilateral multiple pulmonary nodules, both smaller and more significant. The largest was 2.1 cm with moderate bilateral pleural effusion, enlarged mediastinal lymph node (Figure 2).

Bronchial washings and lung biopsy failed to show any acid-fast bacilli or malignancy. Quantiferon gold was also negative. These prompted a video-assisted thoracoscopic surgery (VATS) biopsy with right upper lobe and right lower lobe wedge resection, 10 lymph node resections, and bronchial lavage. Three days post VATS, his dyspnea worsened, and he had hypotension. He needed intubation, ventilation, and pressor support. He also started having a fever and cough. CXR showed right lower lobe consolidation, and complete blood count (CBC) showed leukocytosis with neutrophilia (12,800 cells/mm; erythrocyte sedimentation rate {ESR}, 57mm) rest of the workup, including cultures, were negative. He was started on ceftriaxone, and the fever and cough resolved over the next few days. His biopsy reports were negative for any malignancy and identifiable organisms. The lymph node had rare granulomas, reported as of unknown significance, and abundant mixed dust particles, which were seen better under cross-polarized light and had some silica and silicates and some anthracotic dust. It had patchy organizing pneumonia, especially in the upper lobes superimposed on a background of mixed dust nodules focally becoming confluent with dense parenchymal scarring and abundant mixed dust macules and nodules compatible with mixed dust pneumoconiosis from prior dust exposure. The presence of a 2.1 cm nodule was suggestive of evolving to a later stage of disease, i.e., progressive massive fibrosis. He was started on steroids, anticholinergic and bronchodilator inhalers, which gave symptomatic relief. He was later discharged to follow-up with the pulmonologists.

## Discussion

Pneumoconiosis is due to inhalation of dust, which causes lung inflammation and fibrosis. These diseases typically evolve over several decades, and the pathologic findings in these conditions can resemble those in other fibrotic and granulomatous disorders of the lungs [1]. The toxicity of particulates is related to the nature of the dust and host response [1]. In mixed dust pneumoconiosis (MDP), lung changes are due to simultaneous exposure to silica and less fibrogenic dust, such as iron, silicates, and carbon [4,5], and based on histopathology, MDP is pneumoconiosis showing dust macules and nodules (MDF), with or without silicotic nodules (SN), in an individual with a history of exposure to mixed-dust [6]. In MDP, MDF should outnumber SN in the lung to make a diagnosis on histopathology. Mineralogic analyses can be used for diagnosis if there is no history of exposure [6]. Jobs involving mining, quarrying, foundry, pottery, ceramics, stonemason, agriculture, residential exposure to burning biomass fuel, and using stones to mill grain are associated with MDP [5,6].

Grain dust, pollen, fungi, mites, animal dander, insect pieces, and minerals in the earth's crust are all components of mixed dust in farming. Occupational exposure to dust in agriculture is associated with numerous lung diseases, including chronic obstructive pulmonary disease, asthma, hypersensitivity pneumonia, cancer, and interstitial lung diseases due to exposures to organic and inorganic dust. Up to 15% of farm dust may be silica, and the majority of this is in respirable size [7,8]. MDP is due to exposure to low quantities of silica dust, and as the silica content of inhaled dust rises, lung findings resemble silicosis rather than MDP [5]. MDP development is due to inhalation of 10-18% of free silica [7,8]. Most soils contain a large proportion of crystalline silica (quartz) and silicate minerals, and farmers' exposure to silica and silicates in agricultural dust has been linked to MDP [9,10]. 

Four central interstitial lesions associated with mineral dust exposure are diffuse interstitial fibrosis, macules, nodules, and massive fibrotic lesions, also known as progressive massive fibrosis or complicated pneumoconiosis [10]. Nodules are the most common lesions following exposure to silicate minerals, and they are rounded and firm to palpation and prefer the upper lung zones [11]. Progressive massive fibrosis (PMF) usually occurs against a background of simple pneumoconiosis (macules and nodules) and is formed by the confluence of smaller lesions [12]. PMF is associated with disability and premature death and tends to progress without further exposure. It requires the presence of significant opacity exceeding 1 cm (by x-ray). By pathology standards, the lesion in the histologic section must exceed 2 cm in meeting the PMF definition. In PMF, lesions most commonly occupy the upper lung zone are usually bilateral and are associated with exposure to silica, nonfibrous silicates, and coal mine dust [12]. Our patient had lesions greater than 1 cm in the bilateral upper lung zones; the largest was 2.1 cm. Though MDP is supposed to have a favorable outcome, in this case, it was associated with PMF and cor pulmonale and thus had a poor prognosis.

Cough and dyspnea are symptoms of MDP; radiographic alterations may be seen before the onset of symptoms and altered pulmonary function. The most common radiographic findings in MDP are irregular opacities (ILO, 1980) [6], and the pulmonary function test shows a decreased lung capacity, reduced gas diffusion, and preserved flow rates (forced expiratory volume 1/forced vital capacity {FEV1/FVC}) [13]. Early symptoms may only be exercise-induced dyspnea. Worsening cough and dyspnea at rest are prominent as the condition worsens, and in the later stages, congestive heart failure brought on by cor pulmonale occurs. Tobacco use exacerbates the effects of inhaled dust on the lungs. These patients have a significantly higher chance of developing active tuberculosis (TB) [6,14]. Since lung scarring due to pneumoconiosis can alter the radiographic changes of active TB, it is crucial to check for the disease using microbiologic procedures (such as sputum smear and culture and bronchial washings) [6]. Pneumoconiosis-related lung damage is irreversible [15]. However, steroids, treatment of airflow restriction using bronchodilators, and supplementary oxygen can relieve symptoms and damaging effects of hypoxemia and slow the progression of the illness. Treatment of MDP includes early removal from further exposure, smoking cessation, and severe disease that may require a lung transplant.

## Conclusions

MDP is a type of pneumoconiosis associated with the inhalation of dust with a lower amount of silica in agriculture. Exposure over several decades causes lung inflammation leading to the formation of nodules, macules, and fibrosis mostly in the upper lobes of the lungs. These appear on CXR as irregular opacities larger nodules can coalesce leading to progressive massive fibrosis which has a poor outcome. MDP causes impaired lung functions, cough, dyspnea, and eventually cor pulmonale leading to heart failure. Removal from further exposure is the only treatment and thus early identification is a must. Smoking cessation and active surveillance for TB are necessary. Symptomatic relief can be obtained with corticosteroids, inhaled bronchodilators, and anticholinergics.
